# 1010. Case Study. Wound Healing Against the Odds: Chronic Ulcer Resolution Without Revascularization Using MTX- A Case Report

**DOI:** 10.1093/jbcr/irag033.198

**Published:** 2026-04-06

**Authors:** Crystal C Ukachukwu, Alan Pang

**Affiliations:** Texas Tech University Health Sciences Center, Lubbock, Lubbock, TX; Texas Tech University Health Sciences Center, Lubbock, Lubbock, TX

## Abstract

**Patient Presentation (age range, injury details, relevant history):**

53-year-old female with poorly controlled type 2 diabetes mellitus (HbA1C 16%), hypertension, hyperlipidemia, and peripheral arterial disease. She presented to our facility with chronic, non-healing bilateral lower extremity wounds. Prior to the presentation, she was receiving daily wound care through a home-health service.

**Clinical Challenges:**

-Severe hyperglycemia (blood glucose >700 mg/dl), though without signs of acute metabolic.

decompensation such as diabetic ketoacidosis or hyperosmolar hyperglycemic state.

-Left lower extremity crater ulcer without signs of cellulitis or infection.

-Peripheral arterial disease, as detailed below.

Imaging and diagnostic findings:

• Arterial Duplex Ultrasound: Mild (20 to 49%) stenosis in the left mid superficial.

femoral artery. Multiphasic waveforms were present bilaterally, with minimal flow in the.

distal posterior tibial arteries. Heterogeneous and calcified plaques were noted bilaterally.

• Venous Duplex: No evidence of superficial or deep venous thrombosis.

• Ankle-Brachial and Toe-Brachial Indices (ABI and TBI): Right ABI 1.65, TBI 0.98; Left.

ABI 1.59, TBI 1.19. These elevated ABI values were likely unreliable due to arterial calcifications, which are common in diabetic patients.

**Management Approach:**

Operative intervention:

The patient underwent bilateral lower extremity angiography, which revealed:

• Complete occlusion of the posterior tibial arteries.

• Moderate stenosis in the mid-segment of the right anterior tibial artery.

• Collateral flow via the dorsal pedal arteries, supplying the distal peroneal and common.

plantar arteries.

Attempts at revascularization were unsuccessful.

She subsequently had local wound care treatments. Initial wound coverage was achieved using MTX, a synthetic temporary skin substitute. Once the wound bed was optimized, she underwent successful split-thickness skin autografting, resulting in complete wound healing without complications.

**Outcomes:**

Successful healing of the arterial leg ulcer.

**Lessons Learned:**

Revascularization remains a cornerstone in the management of chronic leg ulcers associated with peripheral arterial disease. However, it is not always achievable due to technical limitations or disease severity. The use of MTX, a foam-based temporary substitute, played a critical role in optimizing the wound bed and facilitating successful graft take.

**Applicability to Practice**:

